# OxyR is required for oxidative stress resistance of the entomopathogenic bacterium Xenorhabdus nematophila and has a minor role during the bacterial interaction with its hosts

**DOI:** 10.1099/mic.0.001481

**Published:** 2024-07-26

**Authors:** Victoria Bientz, Anne Lanois, Nadège Ginibre, Sylvie Pagès, Jean-Claude Ogier, Simon George, Stéphanie Rialle, Julien Brillard

**Affiliations:** 1 DGIMI, INRAE, Univ. Montpellier, Montpellier, France; 2 MGX-Montpellier GenomiX, Univ. Montpellier, CNRS, INSERM, Montpellier, France

**Keywords:** insect, nematode, oxidative stress, OxyR regulon, symbiosis

## Abstract

*Xenorhabdus nematophila* is a Gram-negative bacterium, mutualistically associated with the soil nematode *Steinernema carpocapsae*, and this nemato-bacterial complex is parasitic for a broad spectrum of insects. The transcriptional regulator OxyR is widely conserved in bacteria and activates the transcription of a set of genes that influence cellular defence against oxidative stress. It is also involved in the virulence of several bacterial pathogens. The aim of this study was to identify the *X. nematophila* OxyR regulon and investigate its role in the bacterial life cycle. An *oxyR* mutant was constructed in *X. nematophila* and phenotypically characterized *in vitro* and *in vivo* after reassociation with its nematode partner. OxyR plays a major role during the * X. nematophila* resistance to oxidative stress *in vitro*. Transcriptome analysis allowed the identification of 59 genes differentially regulated in the *oxyR* mutant compared to the parental strain. *In vivo*, the *oxyR* mutant was able to reassociate with the nematode as efficiently as the control strain. These nemato-bacterial complexes harbouring the *oxyR* mutant symbiont were able to rapidly kill the insect larvae in less than 48 h after infestation, suggesting that factors other than OxyR could also allow * X. nematophila* to cope with oxidative stress encountered during this phase of infection in insect. The significantly increased number of offspring of the nemato-bacterial complex when reassociated with the *X. nematophila oxyR* mutant compared to the control strain revealed a potential role of OxyR during this symbiotic stage of the bacterial life cycle.

## Data Availability

The datasets generated and analysed during the current study are available at Gene Expression Omnibus database using the accession number GSE244155.

## Introduction

Bacteria have to cope with various stresses depending on the environmental conditions. Among those, bacterial redox stress can originate from exogenous factors, like abiotic environmental conditions or host response. Exogenous oxidative agents can enter the cells through the membranes by passive diffusion, and such influx can lead to an elevated cellular reactive oxygen species (ROS) level that causes an oxidative stress [1]. In addition, bacterial redox stress can originate from endogenously generated ROS since partially reduced forms of oxygen as well as hydroxyl radicals are formed continuously in aerobic bacterial cells [2,3]. ROS may lead to damage of intracellular macromolecules (DNA, RNA, proteins and lipids) in the cells. In order to avoid harmful effects of these oxidants, bacterial cells have to maintain them below a threshold of toxicity. The main strategy is to produce scavenging enzymes like catalases and peroxidases that degrade ROS. Because of putative damage to DNA, bacteria may also face higher mutation rates [4] and consequently elaborate a response involving activation of DNA repair enzymes [5]. Different redox-responsive transcriptional regulators can control the expression of numerous genes encoding enzymes involved in maintaining redox homeostasis [2,6]. Among them, the importance of OxyR in elaborating a response to mitigate ROS-mediated damages has been described in several bacteria [2].

Conserved in many Gram-negative and -positive bacteria [7,8], OxyR is a transcriptional regulator belonging to the LysR family that functions as a homotetramer [2]. It can act as an activator or as a repressor [7,9]. Depending on the redox state of the bacterial cell, the conformation of OxyR can change due to its disulfide bonds between two conserved cysteine residues, therefore allowing it to bind conserved motifs in the DNA [10,11]. Additional modified forms of OxyR (S-nitrosylation, S-hydroxylation or S-glutathionylation) can occur *in vivo* and produce conformational changes in protein subunits, which yield differential DNA binding and transcriptional activity [12]. Moreover, the OxyR affinity for its DNA target site can also depend on the DNA methylation state, making this regulator one of the few described as involved in bacterial epigenetic regulation [13,14]. Depending on the considered bacterial species, the size of the OxyR regulon can be broadly different, ranging from a few genes (four genes in *Neisseria gonorrhoeae* or five genes in *Shewanella*) to much more (122 genes identified in *Pseudomonas aeruginosa*) [15,17]. In *Escherichia coli*, OxyR induces approximately two dozen genes, including *katG* (encoding a catalase), *ahpCF* [nicotinamide adenine dinucleotide (NADH) peroxidase], *dps* (DNA- and iron-binding protein), *gorA* (GSH reductase) and *grxA* (glutaredoxin), that allow to maintain the redox homeostasis [5]. Besides its function in bacterial adaptation to redox stress *in vitro*, OxyR has often been studied for its *in vivo* role in bacterial virulence, as described in several animal or plant pathogens [18,19]. Studies involving *oxyR* knockout mutants have sometimes been tested in insect hosts: *Yersinia pestis* in *Galleria mellonella* [20], *or P. aeruginosa* in *Drosophila melanogaster* [21]. In these studies, while a role of OxyR in virulence has often been demonstrated, deletion of *oxyR* can also sometimes have no effect on bacterial-host interactions [22].

*Xenorhabdus* are entomopathogenic bacterial members of the *Morganellaceae* family. They are naturally found in a mutualistic symbiosis with soil nematodes belonging to the genus *Steinernema*. These nemato-bacterial complexes are able to infect and consequently kill many insect pests and can be used in biocontrol [23]. *Xenorhabdus* produce antimicrobial compounds in the insect host, which can prevent the multiplication of some soil or insect micro-organisms in the cadaver [24]. During their life cycle, *Xenorhabdus* have to switch between mutualism (within the nematode’s gut) and a pathogenic state (in the insect) [25]. After insect death, the bacteria, together with the nematodes, continue to multiply in the cadaver, during a necrotrophic stage [26]. The *Xenorhabdus–Steinernema* complexes are therefore useful model systems to study mechanisms of pathogenesis and mutualism [25,27]. Gene deletion approaches have revealed the diversity of the mechanisms involved in the interactions between *Xenorhabdus* and its eukaryotic hosts. For instance, various factors were shown to contribute to pathogenesis (such as flagellar regulators [28,29] or peptides [30]), to mutualism (e.g., the Mrx fimbriae [31]; Type 6 Secretion System components [32], lipase [33] and the *nil* genes [34]) or both (e.g., the CpxRA signal transduction system [35] and the global regulator Lrp [36]). Once inside the insect, in addition to its endogenously generated ROS, *Xenorhabdus* likely has to face H_2_O_2_ produced by the host, and presumably other ROS produced by various members of the microbiota [37,38]. Up to now, the importance of oxidative stress encountered during *Xenorhabdus* lifecycle is not known. The role of OxyR in facing oxidative stress seems widely conserved in bacteria, but its diverse contribution to virulence depending on the bacterial pathogens, as well as the high variability in the OxyR regulon composition, has been reported. Here, we investigated the role of OxyR during the lifecycle of *Xenorhabdus*, a bacterial genus that lacks catalase activity [39]. Using a deletion mutant, the genes belonging to the OxyR-regulon were identified, and the OxyR role in *X. nematophila* was investigated *in vitro* during oxidative stress, as well as *in vivo* after reassociation of the bacterial strain with the *Steinernema carpocapsae* nematode.

## Results

### Deletion of the *X. nematophila oxyR* gene impairs growth in oxidative conditions

**Fig. 1.**
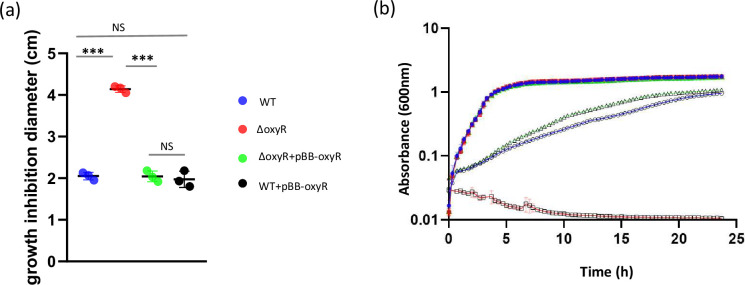
The *X. nematophila oxyR* gene is involved in oxidative stress resistance. (**a**) Growth inhibition of *X. nematophila* F1 WT, ΔoxyR, ΔoxyR+pBB oxyR and WT+pBB oxyR by PQ (1M, 10 µL on a paper disc). Halo size of growth inhibition on LB agar was measured after 24 h. Data are the mean±SD of triplicate independent experiments (*** significant difference, *P*<0.001; NS, no significant difference; t-test). (**b**) Growth of *X. nematophila* F1 WT (blue), ΔoxyR (red) and ΔoxyR+pBB oxyR (green) in oxidative stress condition (paraquat 5 mM, open symbols) or in control (LB, closed symbols). Absorbance at 600 nm was monitored for 24 h at 28 °C. Presented data are the mean values of three independent experiments, and error bars indicate standard deviations.

The XNC3_v3_0510 gene of *X. nematophila* strain F1 (annotated as *oxyR*) displays significant similarity with OxyR gene from several other bacterial species, including two cysteine residues (Cys199 and Cys208), described as involved in the formation of a disulfide bond under oxidizing conditions [40,41]. A putative ribosome binding site sequence (CTGAGG) was found 8 Nt upstream of the ATG start codon. However, no clear OxyR binding site was predicted in the *oxyR* upstream region (www.prodoric.de). An *oxyR* deletion mutant was constructed and exposed to oxidative stress using paraquat (PQ). We first determined that PQ induces the formation of intracellular ROS in both the *X. nematophila* WT and the *oxyR* mutant strains. The ROS level was measured during growth in a standard Luria-Bertani (LB) medium, and no significant difference was observed between the WT and the OxyR mutant strains (Fig. S1, available in the online version of this article). As expected, addition of PQ in the growth medium increased the amount of ROS in the bacterial cells of both strains: 2.9-fold for the WT and 4.6-fold for the OxyR mutant. The *X. nematophila* growth in oxidative condition was then assessed on both solid and liquid media. Using a disc diffusion assay on agar medium, the halo of growth inhibition in the vicinity of the paper disc soaked with PQ (1M) was significantly larger for the *oxyR* mutant strain compared to that observed for the WT strain (*P*<0.05, t-test, Fig. 1a). In LB liquid medium (control condition), the growth curves of both strains had the same shape and overlapped: their slopes were similar during the exponential phase, and they reached the same maximum OD_600nm_ during stationary phase (Fig. 1b). When LB was supplemented with PQ (5 mM), a growth delay was observed for the WT strain, in comparison to LB control condition, revealing a growth impairment by PQ in *X. nematophila*. In the same oxidative condition, no growth was observed for the *oxyR* mutant. These results indicate that *oxyR* is required for *X. nematophila* growth in this oxidative stress condition. Complementation of the mutant strain was performed by introducing a low-copy number plasmid harbouring *oxyR* gene under a constitutively expressed promoter (pBB-oxyR*,* see Table 1). Results indicate that growth was restored by this complementation, to that of the *X. nematophila* WT strain, both on agar and in liquid media (Fig. 1a, b). Interestingly, the introduction of the same plasmid (pBB-oxyR) in the WT strain, which increased the number of *oxyR* copies in the bacterial cell and thus presumably increased the *oxyR* expression level, did not improve the *X. nematophila* resistance to this oxidative stress condition, since no reduction of halo size was observed on agar medium (Fig. 1a). This suggests that the overexpression of *oxyR* does not modify the *X. nematophila* resistance to oxidative stress.

**Table 1.** Strains and plasmids used in this work

Strain or plasmid	Relevant genotype and characteristics*	Reference or source
**Strains**		
*X. nematophila* F1	Wild type	[56]
*E. coli* XL1 blue MRF’	*Δ(mcrA)183 Δ(mcrCB-hsdSMR-mrr)173 endA1 supE44 thi-1 recA1 gyrA96 relA1 lac [F′ proAB lacIqZΔM15 Tn10 (Tetr)]*	Agilent Technologies
*E. coli WM3064*	*thrB1004 pro thi rpsl hsdS lacZΔM15 RP4-1360Δ(araBAD)567 ΔdapA1341::[erm pir (wt)]*	[110]
		
*X. nematophila ΔoxyR*	*ΔoxyR*	This study
*Micrococcus luteus*	Wild type	Pasteur Institute Culture collection, Paris, France
		
**Plasmids**		
pBBR1MCS-5	Cloning vector, Gm^r^	[111]
pBB-D3-gfp	pBBR1MCS-5 plasmid expressing the gfp-gene under the control of the strong and constitutively expressed D3 promoter	[112]
pBB-oxyR	1038 pb PCR fragment (*oxyR* gene) inserted downstream of the P_lac_-promoter in pBBR1MCS-5 plasmid	This study
pHP45-ΩCm	Cm^r^ cassette harbouring plasmid	[113]
pJQ200KS	Mobilizable vector, Gm^r^	[114]
pJQ-ΔoxyR	Region overlapping the *oxyR* gene disrupted by a Cm^r^ cassette and inserted in pJQ200KS plasmid	This study
pPROBE-gfp[AAV]	Plasmid harbouring a promoterless *gfp*-gene	[115]
PoxyR’gfp[AAV]	pPROBE-gfp[AAV] expressing the GFP under the control of the *oxyR* promoter	This study

* Km, kanamycin; Gm, gentamicin; Cm, chloramphenicol.

Several other growth conditions were assessed to compare the phenotypes of the *oxyR* mutant to those of its parental strain. The growth curves of both strains were similar in LB (Fig. 1b). In addition, motility, biofilm formation ability and mutation rate were not significantly different between the two strains (Fig. S2). Other phenotypes usually performed to characterize *Xenorhabdus* strains were also investigated here on the *oxyR* mutant, and no significant difference was observed in comparison to the parental strain for bromothymol blue adsorption on NBTA (nutrient bromothymol blue adsorption medium), antibiotic production and lipase activities (Table S1). Altogether, these findings indicate that among the investigated phenotypes, only the susceptibility to PQ-induced oxidative stress is altered by the *oxyR* deletion in *X. nematophila*.

### Characterization of the *X. nematophila* OxyR regulon

To study the expression of the OxyR regulon in *X. nematophila*, a transcriptomic approach was performed by RNA-seq analysis. WT and *oxyR*-mutant strains were grown in LB in exponential phase, and half of the culture was supplemented with PQ (10 mM) while the second one was used as a control. After 30 min of exposure to this oxidative agent, the cells were harvested for RNA sequencing and analysis. Data revealed a total of 59 genes that were significantly differentially expressed between the two strains (Table 2 and Fig. S3-A). All of them were downregulated in the mutant, except one upregulated in LB condition (XNC3_v3_0986 gene coding for an alkyl hydroperoxide reductase C). Twenty-one genes were differentially expressed at a significant level between the two strains regardless of the growth condition tested (i.e., in both LB control and oxidative condition), while 30 genes were differentially expressed between the two strains only in oxidative-stress condition. The genes belong to various functional groups according to their EGGNOG (Evolutionary Genealogy of Genes: Non-supervised Orthologous Groups) classification, with many of them being phage-related genes, and the most represented category being ‘Metabolism’ (Table 2, Fig. S3-B). The expression level of some of these genes (*argH*, *dps*, *trxB*, *pntA*, *yraJ*, *fimA* and *gor*) was then validated using qRT-PCR (quantitative reverse transcription PCR) analysis. These seven genes were chosen because they were the most differentially expressed between the mutant and the WT strains, according to the RNA-seq data, except for *argH* which was slightly but significantly differentially expressed and was chosen because of its location in the vicinity of *oxyR*. Results confirmed the differential expression of each of these genes between the two strains (Fig. S4-A). A high correlation in the fold-change in expression was observed between the two techniques (RNA-seq and qRT-PCR) (Fig. S4-B).

**Table 2.** Differentially expressed genes identified by RNA-seq between *X. nematophila oxyR* mutant and the WT strain, during growth in oxidative conditions

Label	Gene name	Product	Mutant ΔoxyR/F1 wild-type strain				EGGNOG classification		
			LB		LB +10 mM PQ		OG ID	Process	OG function
			log2 fold change	Adjusted *p*-value	log2 fold change	Adjusted *p*-value			
XNC3_v3_0300	*asnA*	Asparagine synthetase A	−0.32	3.78E-02	−0.45	4.96E-02	05CU9	Metabolism	Asparagine synthetase A
XNC3_v3_0367	–	Conserved protein of unknown function	−0.31	3.78E-02	–	–	063XI	Information storage and processing	Toxic component of a toxin-antitoxin (TA) module. A
XNC3_v3_0507	*argB*	Acetylglutamate kinase	–	–	−0.42	2.89E-02	05CAS	Metabolism	nag kinase
XNC3_v3_0508	*argG*	Argininosuccinate synthase	–	–	−0.51	9.10E-03	05CDH	Metabolism	Citrulline--aspartate ligase
XNC3_v3_0509	*argH*	Argininosuccinate lyase	–	–	−0.73	4.83E-05	05CH7	Metabolism	Arginosuccinase
XNC3_v3_0986	–	Alkyl hydroperoxide reductase C	1.54	4.41E-02	–	–	05D3R	Cellular processes and signalling	Alkyl hydroperoxide reductase
XNC3_v3_1095	–	Conserved protein of unknown function	−0.55	4.96E-02	–	–	01ZBT	Metabolism	Ribosomal protein L11 methyltransferase (PrmA)
XNC3_v3_1410	–	Conserved protein of unknown function	−1.01	1.42E-05	−1.13	2.74E-03	07BCJ	Poorly characterized	na
XNC3_v3_1411	–	DinI-like protein in retron EC67	−0.85	3.43E-05	–	–	06GNW	Poorly characterized	DinI-like protein in retron EC67
XNC3_v3_1412	–	Tail protein X (GpX)	–	–	−1.19	5.44E-05	05YXG	Poorly characterized	Tail protein
XNC3_v3_1413	–	Conserved protein of unknown function	−1.12	5.30E-03	–	–	060YZ	Poorly characterized	na
XNC3_v3_1414	–	Conserved protein of unknown function	−0.81	1.48E-02	−0.84	2.75E-03	05W5F	Poorly characterized	Bacteriophage Rz lysis protein
XNC3_v3_1415	–	Baseplate assembly protein V (GpV)	–	–	−1.17	2.74E-05	05MCM	Poorly characterized	Baseplate assembly protein
XNC3_v3_1416	*w*	Baseplate assembly protein W	–	–	−1.08	9.23E-04	05WAE	Poorly characterized	Baseplate assembly protein
XNC3_v3_1417	*J*	Baseplate assembly protein J	−0.95	8.41E-05	−1.11	1.34E-07	05F1E	Poorly characterized	Baseplate assembly protein
XNC3_v3_1418	–	Tail protein I (GpI)	−1.00	2.52E-04	−1.17	9.41E-06	06WW5	Poorly characterized	Tail protein
XNC3_v3_1419	–	Conserved protein of unknown function	−1.04	6.97E-06	−1.11	2.51E-06	0638C	Poorly characterized	Tail fibre protein
XNC3_v3_1427	–	Putative E14 prophage tail fibre protein (modular protein)	−0.76	2.66E-03	−1.06	5.86E-06	06KCG	Poorly characterized	Phage tail collar domain
XNC3_v3_1428	*xnpS*	Major tail sheath protein XnpS1 of the xenorhabdicin, a R-type bacteriocin	–	–	−1.22	4.08E-06	07QJC	Poorly characterized	Tail sheath protein
XNC3_v3_1429	*xnpT*	Major tail tube protein XnpT1 of the xenorhabdicin, a R-type bacteriocin	–	–	−1.26	3.21E-06	08VRY	Poorly characterized	Tail tube protein
XNC3_v3_1430	–	Conserved protein of unknown function	–	–	−1.17	5.65E-07	05W29	Poorly characterized	Tail protein
XNC3_v3_1431	–	Conserved protein of unknown function	−1.09	4.96E-02	−1.35	8.42E-05	07FHP	Poorly characterized	TR O64312 (EMBL AF063097) (142 aa) fasta scores E()
XNC3_v3_1432	–	Conserved protein of unknown function	–	–	−1.26	4.12E-03	05E3X	Cellular processes and signalling	Lytic transglycosylase catalytic
XNC3_v3_1433	–	Conserved protein of unknown function	−1.02	6.90E-07	−1.06	8.62E-04	05NDM	Poorly characterized	P2 GpU family protein
XNC3_v3_1434	–	Putative phage protein (D protein) (modular protein)	−0.91	4.80E-04	−1.23	3.42E-05	05DAR	Poorly characterized	Late control
XNC3_v3_1435	*ogrK*	Prophage P2 transcriptional activator for bacteriophage P2 late genes	–	–	−1.01	8.62E-04	05ZTC	Information storage and processing	Transcriptional activator, Ogr delta
XNC3_v3_1715	–	Putative alkylphosphonate uptake protein in phosphonate metabolism	–	–	−0.58	4.90E-03	08Z8G	Metabolism	Alkylphosphonate utilization operon protein PhnA
XNC3_v3_1716	*dps*	Fe-binding and storage protein	–	–	−3.24	2.86E-90	05DPV	Metabolism	Protects DNA from oxidative damage by sequestering intracellular Fe(2) ion and storing it in the form of Fe(3)
XNC3_v3_1779	*trxB*	Thioredoxin reductase 1	–	–	−0.37	7.43E-03	05C3M	Cellular processes and signalling	Thioredoxin reductase
XNC3_v3_2487	–	Peptide synthetase	–	–	−0.79	8.08E-05	05C0W	Metabolism	Non-ribosomal peptide synthetase
XNC3_v3_2488	–	Peptide synthetase	–	–	−0.90	5.71E-04	COG1020	Metabolism	Non-ribosomal peptide synthetase
XNC3_v3_2489	*pntB*	Pyridine nucleotide transhydrogenase, beta subunit	–	–	−1.77	4.56E-13	05C15	Metabolism	The transhydrogenation between NADH and NADP is coupled to respiration and ATP hydrolysis and functions as a proton pump across the membrane (by similarity)
XNC3_v3_2490	*pntA*	Pyridine nucleotide transhydrogenase, alpha subunit	–	–	−1.54	2.63E-55	08IIE	Metabolism	The transhydrogenation between NADH and NADP is coupled to respiration and ATP hydrolysis and functions as a proton pump across the membrane (by similarity)
XNC3_v3_2825	–	Conserved protein of unknown function	−2.17	8.56E-28	−1.55	3.37E-19	08YW5	Poorly characterized	Fimbrial protein
XNC3_v3_2826	*yraJ*	Putative outer membrane protein	−2.42	1.74E-33	−1.49	5.21E-18	05CW0	Cellular processes and signalling	Outer membrane usher protein
XNC3_v3_2827	*lpfB*	Putative fimbrial chaperone LpfB	−5.93	5.84E-77	−5.42	4.92E-50	08VCD	Poorly characterized	Chaperone
XNC3_v3_2828	*fimA*	Putative fimbrial subunit (pilin)	−6.41	3.80E-63	−6.04	4.84E-114	05×0I	Cellular processes and signalling	Fimbrial
XNC3_v3_2903	–	Protein of unknown function	−0.65	3.51E-03	–	–	na	Unknown	na
XNC3_v3_3336	–	Phage regulatory protein	−0.74	2.59E-02	–	–	06GH0	Poorly characterized	na
XNC3_v3_3775	–	Conserved protein of unknown function	−0.43	4.45E-03	–	–	na	Unknown	na
XNC3_v3_3934	*mrpA*	Major MR/P fimbrial protein	–	–	−1.68	6.77E-08	05HMV	Cellular processes and signalling	Fimbrial protein
XNC3_v3_4172	*pgi*	Glucosephosphate isomerase	–	–	−0.73	2.22E-05	07QP8	Metabolism	Phosphohexose isomerase
XNC3_v3_4254	–	Conserved protein of unknown function	−0.81	8.39E-03	−0.97	1.15E-06	08KAS	Metabolism	Cholesterol oxidase
XNC3_v3_4306	–	Conserved protein of unknown function	−1.14	8.39E-03	−1.42	1.96E-03	07FAD	Poorly characterized	na
XNC3_v3_4313	–	Putative major capsid protein	–	–	−1.61	3.33E-09	05E9P	Poorly characterized	Phage major capsid protein
XNC3_v3_4314	–	Putative prohead protease	−1.52	5.69E-07	−1.68	3.41E-12	05MAP	Poorly characterized	Phage prohead protease, HK97 family
XNC3_v3_4315	–	Putative head portal protein	−1.30	2.35E-04	−1.59	7.16E-11	05EFC	Cellular processes and signalling	Phage portal protein HK97 family
XNC3_v3_4316	–	Putative head-tail adaptor	–	–	−1.54	2.70E-05	05×0P	Poorly characterized	Head-tail adaptor
XNC3_v3_4317	–	Conserved protein of unknown function	–	–	−1.68	2.22E-05	069NN	Poorly characterized	Phage protein
XNC3_v3_4318	–	HNH endonuclease	−1.47	4.80E-05	−1.39	5.20E-05	05VK6	Cellular processes and signalling	HNH endonuclease
XNC3_v3_4319	–	Conserved protein of unknown function	–	–	−1.38	1.07E-03	0838W	Poorly characterized	Phage terminase small subunit
XNC3_v3_4320	–	Putative terminase large subunit	–	–	−1.13	1.21E-02	05CAR	Information storage and processing	Terminase, large subunit
XNC3_v3_4482	–	Putative head portal protein	−1.23	2.81E-03	−1.04	6.92E-04	05EFC	Cellular processes and signalling	Phage portal protein HK97 family
XNC3_v3_4483	–	Phage phi-C31 gp35-like protein	–	–	−1.16	1.10E-03	05MAP	Poorly characterized	Phage prohead protease, HK97 family
XNC3_v3_4484	–	Phage major capsid protein, HK97	−1.25	1.79E-03	−1.33	3.05E-05	05E9P	Poorly characterized	Phage major capsid protein
XNC3_v3_4491	–	Conserved protein of unknown function	–	–	−0.75	2.12E-02	na	Unknown	na
XNC3_v3_4492	–	Putative phage-related protein	–	–	−0.79	3.60E-02	067 WK	Information storage and processing	Transcriptional regulator
XNC3_v3_4545	–	Conserved protein of unknown function	–	–	−0.56	1.21E-02	08Y1Q	Poorly characterized	na
XNC3_v3_4548	*gor*	Glutathione oxidoreductase	–	–	−2.00	1.01E-46	05DC8	Metabolism	Reductase

*Various shades of graygrey correspond to genes in putative operonic structures. The *oxyR* gene has been removed from this list because the number of reads was biased by the deletion in the ∆oxyR mutant strain.

### The *X. nematophila oxyR* promoter is activated in the *oxyR* mutant

**Fig. 2.**
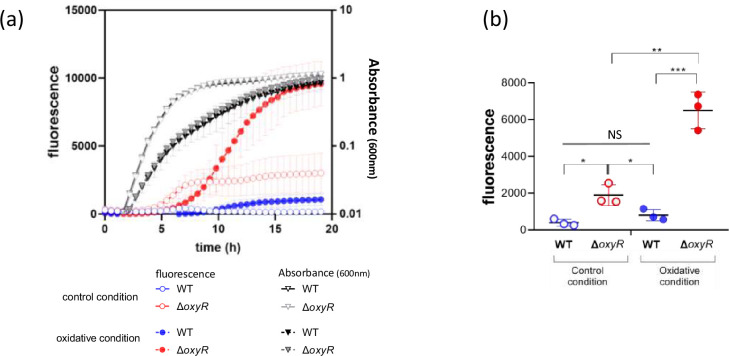
The promoter of the *oxyR* gene is downregulated by OxyR and is activated by PQ. Absorbance at 600 nm (shades of grey) and fluorescence (colours) of WT (blue) and Δ*oxyR* (red) strains carrying the P*_oxyR_gfp*[AAV] plasmid were quantified during growth in control (LB, open symbols) or oxidative stress condition (PQ = 5 mM, closed symbols). (**a**) Fluorescence measurement during growth of strains harbouring the P*_oxyR_gfp*[AAV] plasmid. (**b**) Fluorescence measurement when cells harbouring the P*_oxyR_gfp*[AAV] plasmid reached Abs_600nm_ = 0.5. Error bars represent the standard deviation of the mean (*n*=3). Differences were significant at *, *P*<0.05; **, *P*<0.01; ***, *P*<0.001 (t-tests). NS, no significant difference (*P*=0.1149).

**Fig. 3.**
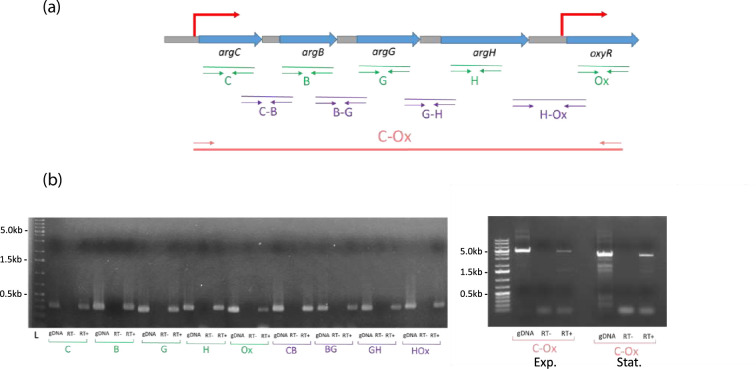
The *X. nematophila oxyR* gene is co-transcribed with genes putatively involved in arginine metabolism. (**a**) Mapping of the primers used on the *argC-oxyR* locus. Thick blue arrows, ORF; grey boxes, intergenic regions. Thin green arrows, primers to amplify intragenic regions; thin purple arrows, primers to amplify intergenic regions. Thin red arrows, primers to amplify the whole locus, from *argC* to *oxyR*. Red broken arrows, putative promoters (schematic representation, not to scale). (**b**) RT-PCR on the *argC-oxyR* locus of *X. nematophila*. Left, primers were designed to amplify the intergenic regions between each gene of this locus (PCR product size ranged between 305 bp and 331 bp). RNA was extracted from *X. nematophila* harvested in exponential phase of growth. Right, primers were designed to amplify the entire locus from *argC* to *oxyR* (PCR product size 4915 bp). RNA was extracted from *X. nematophila* harvested in exponential phase (Exp.) and stationary phase (Stat.) of growth. For each primer couple, the wells correspond to the following: gDNA, positive control (PCR on genomic DNA); RT−, negative control (PCR on RNA without the reverse transcriptase step); RT+, RT-PCR on RNA with the reverse transcriptase step.

In order to investigate the activation of the presumed *oxyR* promoter, the intergenic region between the end of *argH* and the beginning of OxyR ORF (Fig. 3a) on the *X. nematophila* chromosome has cloned upstream the *gfp*[AAV] gene of pPROBE-*gfp*[AAV] vector (Table 1). This construct, pPoxyR’gfp[AAV], was transferred by mating into *X. nematophila* WT and the *oxyR* mutant. During growth of the strains harbouring the pPoxyR’gfp[AAV] transcriptional fusion, the observation of fluorescence confirmed the presence of an activated promoter located in this region (Fig. 2a). The level of fluorescence (mimicking the level of activation of the *oxyR*-promoter) was quantified during growth (Fig. 2a). In the LB-control condition, fluorescence was detected sooner and at a higher level in the *oxyR* mutant in comparison to that observed in the WT strain. During the exponential phase (at OD_600nm_ =0.5), the quantified fluorescence was about fourfold higher in the mutant than in the WT (1895 vs 402 units, Fig. 2b). When grown in oxidative conditions, the activation of the *oxyR* promoter was about eightfold higher in the mutant when compared to that of the WT (6504 vs 813 units, Fig. 2b). These results indicate that disruption of the chromosomal *oxyR* gene increased the activation of the plasmid-encoded *oxyR* promoter, revealing that the *oxyR* gene is negatively autoregulated in *X. nematophila* during these growth conditions. In addition, the activation of the *oxyR* promoter in the presence of PQ was higher for both strains compared to the standard (i.e., LB) condition (Fig. 2), indicating that growth in the oxidative condition activated the *oxyR* promoter.

### The *X. nematophila oxyR* gene is co-transcribed with upstream genes

RNA-seq analysis revealed that three of the four genes encoding proteins putatively involved in arginine metabolism (*argCBGH*), located upstream of *oxyR* in the same orientation (Fig. 3a), were significantly differentially expressed between the *oxyR* mutant and its parental strain, suggesting the existence of an operonic structure that may include the *oxyR* gene. In order to test this hypothesis, RT-PCR was performed on RNA extracted from exponential phase *X. nematophila* cells. For each of the five genes considered (*argCBGH* and *oxyR*), the detection of PCR-amplicons on RNA samples with a reverse transcriptase step indicated that they all were expressed in the tested condition. For each of these genes, the detection of mRNA overlapping its neighbouring gene was observed (Fig. 3b). In addition, using a primer pair mapping from *argC* to *oxyR*, a 4915 bp fragment was observed by RT-PCR amplification (while no amplification was observed in the negative control), indicating that the five genes were co-transcribed in the tested conditions (exponential and stationary phase of growth) and therefore constitute an operon (Fig. 3b). Considering the above-mentioned *oxyR* promoter detected by the observed fluorescence in t strains harbouring pPoxyR’gfp[AAV] (Fig. 2), all these results revealed that (at least) two independent promoters are able to activate *oxyR* transcription, one directly upstream from the *oxyR* gene (located between *argH* and *oxyR*) and one upstream from the *argCBGH-oxyR* operon (Fig. 3a).

### Impact of the *oxyR* deletion on the bacterial lifecycle

The ability of the *X. nematophila oxyR* mutant to colonize its nematode host, *Steinernema carpocapsae*, an entomopathogenic nematode (EPN) was assessed. *G. mellonella* larvae were infected by axenized nematodes [infective juvenile (IJ) stage] and by either the *X. nematophila oxyR* mutant or the WT strain [both harbouring a plasmid allowing the constitutive expression of the GFP (Table 1)]. The bacterial and nematode partners were allowed to multiply in the insect cadavers, and the emerging IJs were collected after 30 days. Results presented in Fig. S5 revealed the presence of GFP-labelled bacteria in the receptacle of all the emerging IJs, indicating that both *X. nematophila* GFP-labelled strains (deleted of the *oxyR* gene or not) were able to colonize the nematodes. This qualitative approach was coupled to the quantification of the amount of bacterial cell per nematode using qPCR. Results revealed no significant difference in the calculated mean number of *X. nematophila* cells per IJ between the two nemato-bacterial complexes (Fig. S6). This indicates that deletion of *oxyR* does not impair the *X. nematophila* ability to re-associate to *S. carpocapsae* in a mutualistic relationship.

**Fig. 4.**
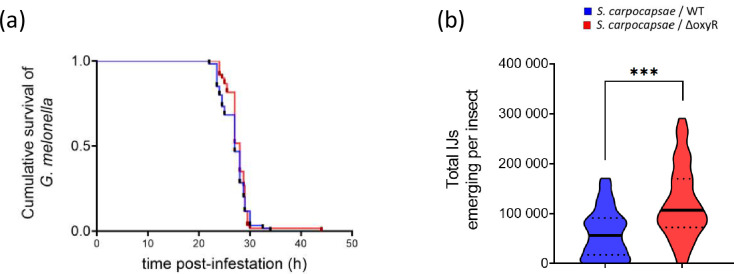
Impact of the *X. nematophila oxyR* deletion after bacterial reassociation with the *S. carpocapsae* nematode. *Galleria mellonella* larvae were infested by 100 *S. carpocapsae* IJs harbouring *X. nematophila* WT (blue) or *oxyR* mutant (red). Data represent three independent experiments, each containing 20 larvae. (a) Survival of *G. mellonella* larvae was monitored over time. The survival curves were not significantly different between the two strains (*P*=0.504 logrank test). (b) Emerging IJs from each *G. mellonella* cadaver 30 days after infestation. The amount of IJs exiting from each cadaver was significantly different between the two strains (t-test with Welch’s correction *P*<0.0001).

In order to determine if life history traits of the nemato-bacterial complexes might be modified by their reassociation with the bacterial strain mutated on the *oxyR* gene, we monitored the survival rate of *G. mellonella* larvae infested by 100 IJs over time. Results presented in Fig. 4a revealed that both nemato-bacterial complexes were pathogenic for the insects as they caused death in less than 48 h. No significant delay in killing *G. mellonella* larvae infested by the *S. carpocapsae*/*X. nematophila* ΔoxyR nemato-bacterial complex was observed throughout the cumulative survival curve, in comparison to that of the *S. carpocapsae*/*X. nematophila* WT nemato-bacterial complex (Fig. 4a). This suggests that OxyR is not required for full virulence in *X. nematophila*. The reproductive success of both these nemato-bacterial complexes was then assessed by quantifying the number of emerging nematodes from insect cadaver after 30 days post-infestation. The amount of IJs emerging from each *G. mellonella* cadaver was significantly higher with the *S. carpocapsae*/*X. nematophila* ΔoxyR nemato-bacterial complex compared to that of the *S. carpocapsae*/*X. nematophila* WT nemato-bacterial complex (Fig. 4b). This suggests that OxyR could play a role during the reproduction phase of the nematodes within the cadaver, affecting the number of offspring.

## Discussion

The role of OxyR in *X. nematophila*, a bacterium mutualistically associated with an EPN, was investigated in the present study by constructing an *oxyR* mutant. While in some bacterial species, *oxyR* deletion can be associated with an impaired growth in control condition (i.e., without particular oxidative stress) compared to their parental strains, such as *Bacteroides fragilis* [42] and *Haemophilus parasuis* [43], and it most often does not affect the *in vitro* growth in others species, nor here in *X. nematophila* during growth in LB. In contrast, when exposed to oxidative conditions, the *X. nematophila oxyR* mutant exhibited a markedly elevated sensitivity compared to the parental WT strain. Such a role has also been described for numerous other bacterial species [2]. The generally conserved function of OxyR is to activate the expression of genes encoding for ROS-scavenging enzymes in response to oxidative agents. The sublethal concentrations of ROS that occur in the environment [44] can be mutagenic for bacteria, even in wild-type strains with functional oxidative stress responses [45,46] indicating an inherent vulnerability in the bacterial ROS defence mechanisms [47]. In mutants that lack a functional *oxyR* gene, a higher mutagenesis rate can be expected, as reported in *E. coli* and *Salmonella typhymurium* [48,50]. However, it is not always the case and may depend on the bacterial strain considered and/or on the tested growth conditions, since a frequency of spontaneous mutation similar between *oxyR* mutants and control strains has also been reported in other studies using the same species [51,52]. The frequency of spontaneous mutations caused by endogenous ROS was investigated in *X. nematophila* after overnight growth in LB, and results revealed that *oxyR* deletion did not significantly cause a difference in the mutation rate, suggesting that no major oxidative stress is encountered and/or that internal mechanisms such as the global response to DNA damage (known as SOS response) are sufficiently efficient to repair DNA damages during this *in vitro* growth condition [5].

Among the other bacterial phenotypes associated with *oxyR* deletion, motility or biofilm formation has been reported in several species, as in *Acidovorax citrulli* [53] where OxyR positively regulates the expression of flagellin (FliC) and type IV pili (PilA) encoding genes. Here, the *X. nematophila oxyR* mutant exhibited no significant difference compared to the parental strain in its motility or biofilm-forming ability, as observed elsewhere [54,55].

OxyR is a transcription factor that regulates several genes involved in anti-oxidative stress, such as catalase [2]. The description of the whole OxyR regulon was performed using an RNA-seq approach in *X. nematophila* in LB or in PQ-treated condition. Among the genes for which a differential level of expression was observed in the mutant compared to the parental strain, none display similarity with known catalase-encoding genes. This is in agreement with the fact that, in the *Xenorhabdus* genera, no catalase activity has been described [39], and no catalase-encoding gene has been identified [56]. However, some genes shown to belong to the OxyR regulon of other gamma-proteobacteria [57], such as *dps*, *gorA*, *trxB* and *pntA,* were also identified here. Some of them encode proteins with ROS-scavenging activities (NADH peroxidase; GSH reductase; a ferritin that lowers the availability of iron for Fenton reactions) [58,59]. The gene *ahpC*, coding an alkylhydroperoxide reductase subunit C, is known to be activated by OxyR during oxidative conditions in many bacteria [7,59]. Surprisingly, our result suggests that expression of *ahpC* in *X. nematophila* is repressed by OxyR during non-oxidative stress condition, since this gene was the only one that was upregulated (1.5 fold) in the *oxyR* mutant compared to the WT in our conditions (during non-oxidative stress condition only). In the obligate anaerobic bacteria responsible for periodontitis, *Tannerella forsythia* [60] and *Porphyromonas gingivalis* [61], both being catalase negative similar as *X. nematophila*, *ahpC* is positively expressed by OxyR and this may compensate for the absence of catalase activity in peroxide detoxification. In *Burkholderia thailandensis*, *ahpC* was also shown to be negatively regulated by OxyR, while still helping to protect the bacteria from ROS [62]. Here, OxyR might indirectly regulate *ahpC* through the involvement of other regulatory proteins, leading to repression under non-oxidative conditions. Further studies are needed to elucidate the exact mechanisms underlying the *ahpC* regulation in *X. nematophila*.

Interestingly, a highly differentially expressed gene identified here during both oxidative-condition and control-condition, *fimA* encoding for a fimbria, was also found to belong to the OxyR regulon in *P. gingivalis* [61]. Fimbria are involved in the attachment and colonization of host tissues [63], and this finding may reveal a role of OxyR in the ability of *X. nematophila* to adhere to and colonize the nematode and/or insect’s tissues.

A high correlation in the fold-change expression was observed between the qRT-PCR and RNA-seq techniques. Altogether, these results enhance confidence in the list of the differentially expressed genes identified in this study, confirming the OxyR regulatory function in *X. nematophila*. Most differentially expressed genes were downregulated in the *X. nematophila oxyR* mutant, indicating a transcriptional activator role of OxyR in the tested conditions. Here, 59 genes were identified as differentially regulated in the *X. nematophila oxyR* mutant. This number is in the range of the OxyR regulon size described elsewhere, since it can vary significantly depending on bacterial species considered (from four genes in *Neisseria gonorrhoeae* [15] to hundreds of genes in *H. parasuis* [43]. The described sizes of OxyR regulon may also vary depending on the growth conditions in which the sampling for the analysis has been done, because the growth phase significantly contributes to bacterial resistance to a variety of stress conditions, including exposure to oxidative agents [64,65].

Among the differentially expressed genes, those localized in the vicinity of *oxyR* and encoding putative enzymes involved in arginine metabolism were identified. RT-PCR experiments revealed that these genes were co-transcribed with *oxyR* and therefore belong to the same operon. A similar observation was made in *E. coli* [66]. The transcriptional fusion with the *gfp* reporter gene revealed a promoter located immediately upstream of *oxyR*. Our results revealed that the disruption of the chromosomal *oxyR* gene increased the activation of the plasmid-encoded *oxyR* promoter, suggesting that the *oxyR* gene is negatively autoregulated in *X. nematophila*. Whether this regulation is direct or not remains to be investigated, since this observation could potentially be explained by OxyR controlling another regulator, which then would activate the cloned promoter fragment of the pPoxyR’gfp[AAV] construct. Anyway, all these results indicate that at least two promoters can control *oxyR* transcription in *X. nematophila*. Interestingly, this synteny is conserved in the closely related genus *Photorhabdus*, but also in other gamma-proteobacteria such as *E. coli* [67]. Whether a similar co-transcription between *oxyR* and arginine metabolism-encoding genes also exists in these bacterial species and understanding the function of such co-transcription in the bacterial physiology remain to be investigated. Our RNA-seq data indicate that the *argCBGH* operon is overexpressed after addition of PQ when compared to control condition, for both the WT and the *oxyR* mutant strain. This reveals that the distal *oxyR* promoter (upstream of *argC*) is activated in oxidative condition in an OxyR-independent manner. Assessing whether differential regulation in oxidative conditions is similar or not in other bacterial species will require further studies.

Besides its *in vitro* involvement in bacterial adaptation to oxidative stress, several studies involving knockout mutants of bacterial pathogens revealed the contribution of OxyR in virulence. This was shown in mammalian pathogens, such as *E. coli* [19], *Klebsiella pneumoniae* [68], *B. fragilis* [42], *Francisella tularensis* [69] or *P. aeruginosa* [70] in various animal models, including insect hosts [20,21]. OxyR can also contribute to virulence of plant pathogens such as *Ralstonia solanacearum* [18], *Agrobacterium tumefaciens* [71], *Xanthomonas campestris* [72] and *Xanthomanas oryzae* [73]. In addition, OxyR can also contribute in surviving the challenge of ROS encountered by some bacterial pathogens, during their free-living stages in the environment [58], such as *Vibrio cholerae* in seawater [74] or *R. solanacearum* in soil or water [75].

However, to our knowledge, there are no reports regarding the role of OxyR during a bacterial mutualistic association with its host. Because *X. nematophila* bacteria are specific symbionts of the nematode *S. carpocapsae*, we reassociated the *oxyR* mutant strain with its partner nematode. The pathogenicity of this successfully formed nemato-bacterial complex was then assessed in insect larvae, as during its natural life-cycle. The *oxyR* deletion in *X. nematophila* did not reveal a significant change in the ability of the nemato-bacterial complex to kill *G. mellonella* larvae. Deletion of *oxyR* has also been shown in other bacterial species to have no effect in virulence, as in *Corynebacterium diphtheriae* [76], *Neisseria gonorrhoeae* [15] or *Mycobacterium maritimus* [22].

Because we have shown that *X. nematophila oxyR* mutant displayed impaired growth during *in vitro* oxidative conditions, these *in vivo* results therefore suggest that additional factors, other than the *X. nematophila* OxyR, may help the nemato-bacterial complex to cope with oxidative stress encountered during the interaction of these multiple organisms (insect-nematode-microbiota). Indeed, oxidative stress can be an important immune mechanism used by insect to resist microbial infections (as shown in * G. mellonella* after infection by *Salmonella enterica* [37]). A possible explanation is the efficiency of the nemato-bacterial complex to avoid the effects of the insect host immune system: *X. nematophila* produces several cytolysines that may prevent phagocytosis and nodulation processes [77], as well as metabolites able to inhibit the phenoloxidase cascade [78], and the cognate nematode is also able to compromise host immunity [79]. The role played by the whole bacterial microbiota should also be considered [80]. An additional explanation could be alternative regulators that may allow to elicit an anti-oxidative response in *X. nematophila* during the infectious process. In bacteria, the defence system used to counter oxidative stress can be orchestrated by several transcriptional factors with overlapping functions, including OxyR [81,82]. The importance of such additional regulators (SoxS is found in the *X. nematophila* genome for instance) when coping with oxidative stress *in vivo* remains to be explored. In addition, we cannot rule out the existence of an host compound that would directly interact with the OxyR protein, as described for the plant pathogen *Xanthomonas* [83], therefore leading to no phenotypic differences between the WT and the *oxyR* mutant strains.

In our study, this tripartite experiment also allowed us to investigate the impact of *oxyR* deletion during *X. nematophila* mutualistic interaction with the nematode. As mentioned above, both the WT and the mutant bacterial strains were able to reassociate with *S. carpocapsae*, with a similar level of colonization, revealing that they succeeded in supporting the nematode development. Interestingly, since a slightly increased reproductive success (measured as the number of offspring emerging from the insect cadaver) was observed for the nemato-bacterial complex harbouring the *X. nematophila oxyR* mutant, our results suggest that OxyR in *X. nematophila* may play a role in the nematode’s fitness in the *G. mellonella* model. However, we cannot rule out that this phenotype is linked to OxyR in an indirect manner. An additional experiment using axenized EPNs reassociated to a Δ*oxyR* complemented strain would be required to confirm if the WT phenotype is restored *in vivo*. Given that trophic analyses demonstrate that the nematode feeds on the bacteria and that the bacteria are responsible for consumption of the insect [84], this may suggest a slightly higher availability of the nutrients for the nematode when feeding on the *X. nematophila oxyR* mutant. Interestingly, a tween-specific lipase was also shown to play a role in supporting production of nematode progeny in *G. mellonella* [33], emphasizing the fact that nutrient availability in insect cadaver is important for EPN reproductive success. The observation of several bacteriocin or phage-related genes differentially regulated in the *oxyR* mutant compared to the WT in our RNA-seq data has been observed elsewhere [85,87]. Considering the EPN as holobionts, future studies may also consider oxidative stress, since changes in OxyR regulation of phage-related genes could impact the associated microbiota composition and possibly the EPN life-cycle [88]. Studying all these interactions may provide new insights to better understand their efficiency in killing insect pests and therefore enhance their use in biocontrol.

In conclusion, we report here that *oxyR* is required for *X. nematophila* optimal growth in *in vitro* ROS-enriched environments but not during interactions with its eukaryotic hosts. This bacterial species lacks a catalase-encoding gene, but several genes that presumably contribute to the oxidative stress response could be identified as members of the OxyR regulon. Altogether, our results suggest that factors other than OxyR could allow *X. nematophila* to cope with oxidative stress encountered during the insect infection. Future studies should consider the importance of OxyR during bacterial mutualistic interactions with their host.

## Methods

### Strains and growth conditions

The strains used in this study are listed in Table 1. The *X. nematophila* F1 and *E. coli* bacterial strains were routinely grown in LB medium with a 180 r.p.m. agitation at 28 °C or 37 °C, respectively. As required, antibiotic concentrations used for bacterial selection were chloramphenicol 15 μg mL^−1^, gentamycin at 15 μg mL^−1^, rifampicin at 50 μg mL^−1^ and kanamycin at 20 μg mL^−1^. The oxidative agent PQ (also known as methyl viologen dichloride hydrate, Sigma-Aldrich) was added when required, at concentrations to either abolish the growth or to allow a remaining (impaired) growth of the mutant strain, and was based on similar studies in other bacterial species [15].

Bromothymol blue adsorption; antibiotic production against *Micrococcus luteus*; lipase activity on Tween 20, 40, 60, 80 and 85; and hemolytic activity were determined as previously described [89; 90]. For motility assays, agar plates were prepared with LB broth supplemented with 0.35 % agar and inoculated using 5 µL of cells grown in exponential phase (OD_600nm_ = 0.8), as previously described [91]. The diameter of the halo size of swimming motility was measured 20 h after incubation. Data from three independent experiments (with 10 plates used in each condition) were analysed using the Wilcoxon test. The spontaneous mutation rate was assessed by quantifying the emergence of rifampicin-resistant c.f.u.s, as previously described [92]. Briefly, strains were grown overnight in 40 mL of LB medium to reach a population above 1×10^9^ c.f.u. mL^−1^. Bacterial cells were serially diluted and plated on GNO +/-rifampicin (50 µg mL^−1^). The mutation rate was calculated as the rifampicin-resistant population divided by the total population. Data from three independent experiments were compared and analysed by t-test. Biofilm formation was monitored as previously described [92]. Briefly, 5 mL of LB medium in glass tubes was inoculated with an overnight culture and incubated for 12 days at 28 °C in static conditions. The tubes were then rinsed with PBS before the addition of 7 mL of crystal violet solution at 0.01% (in PBS) to stain the biofilms for 15 min. Biofilms were rinsed with PBS and then dissolved for 3 h in 7 mL of ethanol. The OD_570 nm_ measurement allowed the quantification of the biofilm-associated crystal violet. Data from three independent experiments with replicates (totalizing 30 tubes for each strain) were analysed using a Wilcoxon test.

Resistance to oxidative stress was quantified using PQ (paraquat dichloride hydrate, Sigma), as follows. A 6 mm paper disc (Millipore) soaked with 10 µL of PQ (1M) was placed on LB agar medium previously inoculated with 1 mL of exponential phase-grown bacterial cells (OD_600nm_ = 0.6). Halo size of growth inhibition was measured after 24 h. For each of the three independent experiments, technical triplicates were performed. Presented data were analysed using a Student’s t-test. In liquid medium, growth of *X. nematophila* strains in LB, or LB supplemented to 5 mM PQ, was monitored with a TECAN automated turbidimetric system (Infinite M200 TECAN), as previously described [92]. Experiments were performed on three independent biological replicates for each strain.

Cellular ROS level was quantified by fluorescence spectroscopy as previously described [93]. Briefly, *X. nematophila* cells were grown in exponential phase. At OD_600nm_ = 0.3, PQ (2.5 mM) was added (or not for the control condition), and growth was resumed for 30 min. Cells were then harvested by centrifugation (2 min at 13 000 r.p.m.) and washed twice in PBS buffer, followed by an incubation step of 30 min with DCFH-DA (2,7-dichlorofluorescein-diacetate at 100 µM, D6883, Sigma-Aldrich). Fluorescence was measured using a microplate reader (excitation wavelength 485 nm, emission wavelength 535 nm). Each independent experiment was performed in technical triplicate. The presented data are from three independent experiments and were analysed using t-test.

*X. nematophila* wild-type and ∆oxyR strains carrying pPoxyR’gfp[AAV] (Table 1) were cultured in black-sided, clear-bottomed 96-well plates (Greiner) as follows. For each well, 10 µL of an overnight culture diluted 1/40 was added to 190 µL of LB supplemented with kanamycin, with or without PQ as required. The plates were incubated in an Infinite M200 microplate reader (Tecan) at 28 °C with shaking. The OD_600nm_ and the GFP fluorescence (excitation 485 nm, emission 535 nm) were monitored every 30 min during 20 h. Three biological independent replicates were carried out for each condition.

### Cloning and molecular biology

Chromosomal DNA was extracted from bacterial cells using the QIAamp DNA Mini kit (Qiagen, Courtaboeuf, France). The extraction of plasmid DNA from *E. coli* was performed using the GenElute™HP Plasmid miniprep purification kit as recommended by the manufacturer (Sigma, Saint-Quentin-Fallavier, France). Restriction enzymes and T4 DNA ligase were used as recommended by the manufacturers (New England Biolabs, Evry, France and Promega, La Farlede, France, respectively). Oligonucleotide primers sequences were designed using the Primer3 software [94]. They were synthesized by Integrated DNA Technologies (IDT, Leuven, Belgium) and are listed in Table S2. PCR was performed in a T100 thermal cycler (Biorad, Marnes-la-Coquette, France) using the iProof high-fidelity DNA polymerase (Biorad). Amplified DNA fragments were purified using a PCR purification kit (Ozyme, St Cyr LEcole, France) and separated on 0.7 % agarose gels after digestion as previously described [95]. Digested DNA fragments were extracted from agarose gels with a centrifugal filter device (Ozyme). All constructions were confirmed by DNA sequencing (Eurofins Genomics, Ebersberg, Germany).

Construction of the *oxyR* mutant was performed as follows. DNA fragments of the *oxyR* (XNC3_v3_0510) upstream (650 pb) and downstream (630 bp) regions were PCR-amplified using the primer pairs upF-oxyR-Pst/upR-oxyR-Sal and dnF-oxyR-Sac/dnR-oxyR-Xba, respectively (Table S2). PCR products were digested with PstI/SalI and SacI/XbaI using the primer-incorporated restriction sites. In parallel, a Cm^r^ cassette was PCR amplified from pHP45-ΩCm (Table 1), using the primer pairs Cm-F/Cm-R (Table S2) and digested with SalI/SacI, as previously described [96]. The three digested DNA fragments were purified, ligated in PstI/XbaI-digested pJQ200KS (Table 1) and introduced by electroporation in *E. coli* XL1. The resulting pJQ-ΔoxyR plasmid was transferred in the *E. coli* donor strain WM3064 and then introduced in *X. nematophila* by conjugative mating as previously described [28]. Four independent transconjugant clones were isolated and subjected to allelic exchange in LB at 28 °C, following the protocol routinely used in the laboratory [96,98]. Clones of *oxyR* mutants were positively selected on sucrose plate and verified by PCR using VerifDoxyR-F and VerifDoxyR-R primers (Table S2) and sequencing. Furthermore, no polar effect of the deletion was observed according to the RNA-seq data showing no difference in expression of the *oxyR* surrounding genes in LB-control condition, between the WT and the mutant strain (Table 2).

Cloning the *X. nematophila oxyR* gene under P*_lac_* promoter was performed as follows. A 1038 bp DNA fragment was PCR amplified using two primers mapping upstream and downstream (Cp-oxyR -F/Cp-oxyR-R, respectively, Table S2) of the *oxyR* gene, using the following cycling conditions: 95 °C, 10 s; 56 °C, 30 s; 72 °C, 45 s for 35 cycles. It was then digested according to the endonuclease sites introduced in the primers (EcoRI/BamHI) and was inserted between the corresponding sites of the low-copy plasmid pBBR1MCS-5 (Table 1) downstream of the P*_lac_* promoter. The recombinant plasmid (pBB-oxyR) was introduced in *E. coli* WM3064 donor strain and then transferred in *X. nematophila* as described above. Transconjugants harbouring the pBBR1MCS-5 empty plasmid were used as a control. The construction of a transcriptional fusion between the upstream *oxyR* region and the *gfp*-reporter gene was performed as follows: DNA fragment harbouring putative promoter was amplified by PCR from *X. nematophila* F1 genomic DNA using the primer pairs PoxyR-F-Xba/PoxyR-R-Eco (Table S2). The PCR products were purified and digested with *Eco*RI and *XbaI* and inserted into the corresponding sites of pPROBE-*gfp[*AAV], a plasmid encoding a promoterless *gfp*-reporter gene (Table 1). The plasmids were transferred to *X. nematophila* by conjugative mating as described above.

### RNA extractions, sequencing and amplification assays

*X. nematophila* wild-type and ∆*oxyR* strains were grown in 20 mL LB at 28 °C with shaking, after inoculation by an overnight growth culture at 0.5 %. When OD_600nm_ reached 0.3, the culture was split into two parts, and one of them was supplemented with PQ (10 mM) after which growth was immediately resumed for 30 min to generate an oxidative stress. The second one was used as a control. Four millilitres were then mixed with 8 mL of RNA-protect solution according to the manufacturer’s instructions (Qiagen). Total RNA extraction was performed using RNeasy miniprep Kit (Qiagen), with an additional incubation step with TURBO DNase (Invitrogen) as previously described [99]. The quantity and quality of RNA were assessed with an Agilent 2100 Bioanalyzer with the RNA 6000 Nano LabChip kit. The lack of DNA contamination was controlled by carrying out a PCR using 63bis and 153Rev primers mapping the 16S region (Table S2) on each RNA preparation. Nine independent cultures with cells harvested in exponential phase were performed for each strain and each growth condition.

For the RNA sequencing, equal amounts of total RNA from three independent samples per strain and per condition were pooled together to generate one final biological RNA sample per strain. Thus, from the initial nine independent RNA samples per strain and per condition, three final RNA samples were generated for each strain and subsequently treated as follows prior sequencing. MicrobExpress Bacterial mRNA Enrichment Kit (Ambion) was used to remove ribosomal RNA from 4 µg of total RNA according to the manufacturer’s instructions.

RNA-seq libraries were prepared using the Truseq stranded mRNA sample prep kit (Illumina, San Diego, CA, USA) according to the manufacturer’s instructions. Briefly, the RNAs, previously depleted in rRNA, were fragmented using divalent cations at elevated temperature and reverse transcribed using random hexamers, SuperScript II (Thermo Fisher Scientific, Carlsbad, CA) and actinomycin D. Deoxy-TTP was replaced by dUTP during the second strand synthesis to prevent its amplification by PCR. Double stranded cDNAs were adenylated at their 3' ends and ligated to Illumina’s adapters containing unique dual indexes (UDIs). Ligated cDNAs were PCR amplified for 15 cycles PCR and the PCR products were purified using AMPure XP Beads (Beckman Coulter Genomics, Brea, CA, USA). The size distribution of the resulting libraries was monitored using a Fragment Analyzer (Agilent Technologies, Santa Clara, CA, USA), and the libraries were quantified using the KAPA Library quantification kit (Roche, Basel, Switzerland). The libraries were denatured with NaOH, neutralized with Tris-HCl and diluted to 300 pM. Clustering and sequencing were performed on a NovaSeq 6000 (Illumina, San Diego, CA, USA) using the single-end 100 nt protocol on one lane of a flow cell SP.

Image analyses and base calling were performed using the NovaSeq Control Software and the Real-Time Analysis component (Illumina, San Diego, CA, USA). Demultiplexing was performed using Illumina’s conversion software (bcl2fastq 2.20). The quality of the raw data was assessed using FastQC (v0.11.8) from the Babraham Institute and the Illumina software SAV (Sequencing Analysis Viewer). FastqScreen (v0.14.0) was used to identify potential contamination.

Reads were aligned to the reference genome (*X. nematophila* XENF1.2) with BWA-MEM/BWA-ALN (v0.7.17-r1188) [100]. Reads with MAPQ scores less than three were filtered out using SAMtools view (v1.9, options -F 0×904 q 3) to eliminate the reads that were likely to align to multiple positions. SAMtools was also used to sort and index the alignment files. Then, gene counting was performed with featureCounts v2.0.0 [101] using a gff file downloaded on 27 July 2020 from the MaGe platform (https://mage.genoscope.cns.fr) [102]. As the data are from a strand-specific assay, the reads have to be mapped to the opposite strand of the gene (-s two option). Before statistical analysis, genes with less than 15 reads (cumulating all the analysed samples) were filtered out.

Differentially expressed genes were identified using the Bioconductor [103] package DESeq2 v1.26.0 [104] (R version 3.6.2). Data were normalized using the DESeq2 normalization method. Genes with adjusted *p*-value below 5 % (according to the FDR method from Benjamini-Hochberg) were called differentially expressed.

The complete dataset from this study has been deposited in NCBI’s Gene Expression Omnibus database, under accession number GSE244155.

### RT-PCR

RNA from *X. nematophila* cells grown in exponential phase (OD_600nm_ =0.4) or stationary phase (OD_600nm_=2.5) was extracted as described above. Lack of DNA contamination was controlled by carrying out a PCR on 16S gene using 63bis and 153Rev primers (Table S2) on each RNA preparation as previously described [90]. RT-PCR reactions were performed as follows. A total of 500 ng of total RNA was denatured at 70 °C for 5 min. Conversion into cDNA was carried out using random hexamers (C1181, Promega) and SuperScript II reverse transcriptase (Invitrogen), for 10 min at 25 °C, then 50 min at 42 °C and finally 15 min at 70 °C. PCR amplifications on the cDNA were carried out with a Go-Taq DNA polymerase (Promega) using primers listed in Table S2. The following amplification conditions were applied: 3 min at 95 °C, 30 s at 55 °C and 15 min at 72 °C, for 35 cycles. Positive controls were performed using *X. nematophila* F1 genomic DNA as template. RNA samples that have not undergone the reverse transcriptase treatment were used as negative controls. The PCR products were loaded on an agarose gel followed by visualization on a UV transilluminator.

### RT-qPCR analysis

Quantitative reverse transcription-PCR (RT-qPCR) was carried out as previously described [90] on seven genes selected because they were highly differentially expressed between the WT and *oxyR* mutant strains according to the RNA-seq analysis. Briefly, RNA samples from three biological replicates for each strain and each growth condition were used for cDNA synthesis as described above. The SuperScript II reverse transcriptase (Invitrogen) was used on 0.5 µg of total RNA with random hexamers (100 ng·µL^−1^; Roche Diagnostics). qPCR analyses were performed using SensiFAST™ SYBR No-ROX Kit (Bioline) with 0.5 µL of cDNA synthesis mixture (diluted 1:50) and specific gene primers at 1 µM (Table S2). The reactions were performed in duplicate at 95 °C for 2 min, followed by 45 cycles at 95 °C for 5 s, 61 °C for 25 s and 70 °C for 15 s and monitored in the LightCycler 480 system (Roche). Melting curves were analysed and always contained a single peak. The data were analysed with the REST software 2009 [105] using the pair-wise fixed randomization test with 5000 permutations. Data are presented as a ratio with respect to the reference housekeeping gene *recA*, as previously described [90].

#### Bacterial reassociation with axenic nematodes

*S.carpocapsae* (strain SK27) axenic nematodes were produced as previously described [106]. Briefly, 4–6 days after insect infestation with L3 (infective juvenile, IJ), gravid female nematodes were collected from *G. mellonella* cadaver. Eggs were extracted from washed female by crushing. The intact eggs (checked by microscopic observation) were placed on sterile liver-agar supplemented with antibiotics. After 30 days in a sterile, dark and humid environment, axenic IJs were collected. The absence of *Xenorhabdus* in the axenized nematodes was verified by DNA extraction followed by PCR amplification with *Xenorhabdus*-specific primers (Xeno_F/Xeno_R, Table S2) that target a region of the XNC1_0073 gene encoding a putative TonB-dependent haem receptor [107].

*X. nematophila* WT and ∆*oxyR* strains harbouring a plasmid expressing GFP under the control of a constitutive promoter (pBB-D3-gfp, Table 1) were obtained using the mating protocol described above. They were grown in LB until an OD_600nm_ of 0.6, harvested and washed in fresh LB. Approximately 10**^7^** c.f.u. in 20 µL LB supplemented with kanamycin were injected into *G. mellonella* larvae (five larvae for each strain) in order to initiate the *in vivo* bacterial growth. After 6 h, insects were still alive and were infested by axenic IJs, in order to allow a multiplication of the nematodes inside the infected insect larvae. The infestations were performed by pipetting 100 axenic IJs onto each immobilized *G. mellonella* larvae in 1.5 mL Eppendorf tubes, as previously described [108]. The new generation of IJs that emerged was collected, and the presence of *X. nematophila* WT or ΔoxyR expressing the GFP in the IJ’s receptacle was confirmed under a fluorescence microscope (Fig. S5). The nematodes were stored in the dark at 9 °C.

#### Nemato-bacterial infestation

Twenty *G. mellonella* larvae were individually infested by 100 IJs and were then incubated in the dark at 23 °C. Three independent replicates were performed (totalizing 60 insect larvae for each nemato-bacterial complex). Insect survival was monitored over time to evaluate the virulence-related properties of each nemato-bacterial complex up to 45 h after infestation. Data were analysed in GraphPad Prism 8 software using the log-rank method with 95 % CI. Dead larvae were then placed individually on white traps [109]. After up to 30 days, the emergence of nematodes was observed for all dead larvae. To evaluate the fitness of the nematodes, the reproductive success (defined as the number of IJs that emerged from each insect larvae) was quantified after 30 days of infestation. As the reproductive success of nematodes is neither normally distributed nor normalizable, data were analysed using a Mann–Whitney test as previously described [108].

### Quantification of *Xenorhabdus* smbiotically associated with *S. carpocapsae*

DNA was extracted from *S. carpocapsae* SK27 ApoIII/WT and ApoIII/∆*oxyR (IJs*) as previously described [80], with the following modifications. First, 100 IJs harbouring the Gfp-labelled *X. nematophila* strains were individually collected with a sterile pipette, transferred into screw-top microtubes (2 mL) and immediately frozen at − 80 °C. A heat stress (20 min incubation at +80 °C after their removal from the freezer) allowed to weaken the IJs’ double cuticle. Then, six 2-mm glass beads were added in each tube, and nematodes were mechanically disrupted using the FastPrep instrument (MP Biomedicals, Illkirch-Graffenstaden, France) for 3 cycles of 7 m/s of 40 s. One hundred microlitres of Quick Extract lysis solution (Bacterial DNA Extraction Kit from Epi-centre, USA), 1 µL of Ready-Lyse Lysozyme Solution (Epi-centre, USA) and 20 µL EDTA (0.5 M, pH 8) were added, and complete lysis of both nematodes and prokaryotic cells was obtained by incubation at room temperature for 90 min. Ten microlitres of Proteinase K (20 mg mL^−1^) was added followed by incubation at 55 °C with shaking until the solution was cleared. RNA was eliminated with 10 µL of RNaseA at 20 mg mL^−1^ (Invitrogen PureLink™ RNaseA, France) for 15 min at 37 °C. Proteins were precipitated by adding 100 µL of protein precipitation solution (Kit Wizard, Promega, France) followed by an incubation time of at least 5 min on ice; the samples were then centrifuged 5 min at 17 000 g at 4 °C, and the supernatant was collected. DNA was precipitated with isopropanol, resuspended in 50 µL ultrapure water and stored at −20 °C. The amount of *Xenorhabdus* in each IJ was then quantified using *Xenorhabdus*-specific primers (Xeno_F/Xeno_R, Table S2) as previously described [107]. Axenic nematode without *Xenorhabdus* were used as a control. Data from three independent samples were compared using the t-test with Welch’s correction.

## supplementary material

10.1099/mic.0.001481 Uncited Supplementary Material 1.

## Abbreviations

EGGNOG: evolutionary genealogy of genes: Non-supervised Orthologous Groups
EPN: entomopathogenic nematode
IJ: infective juvenile
LB: Luria-Bertani
NADH: nicotinamide adenine dinucleotide
NBTA: nutrient bromothymol blue adsorption medium
PQ: paraquat
ROS: reactive oxygen species
RT-qPCR: quantitative reverse transcription-PCR
TA: toxin-antitoxin
UDI: unique dual index
