# Supplementary material for: OxyR is required for oxidative stress resistance of the entomopathogenic bacterium Xenorhabdus nematophila and has a minor role during the bacterial interaction with its hosts
Source: Microbiology (Reading). 2024 Jul 26;170(7):001481. doi: 10.1099/mic.0.001481 (PMC11281485; doi:10.1099/mic.0.001481)
Supplement: Uncited Supplementary Material 1. [file mic-170-01481-s001.pdf]

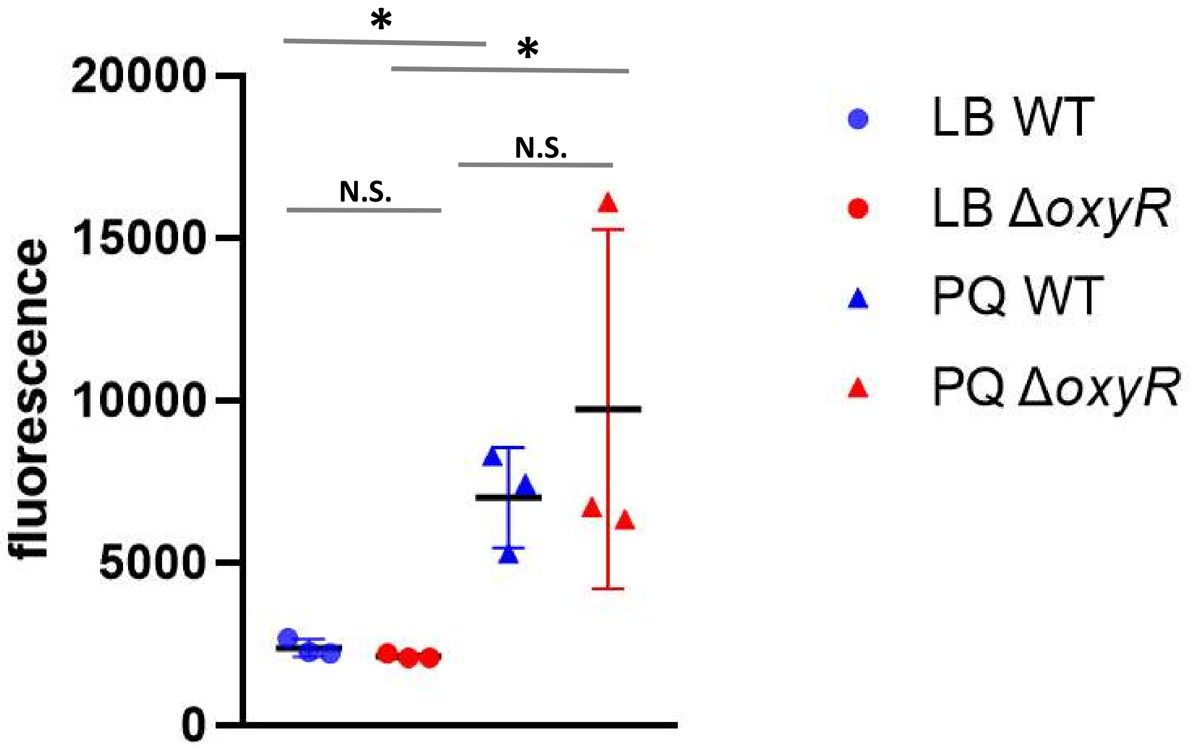

Fig. Supp 1. Paraquat induces ROS in both WT and *oxyR* mutant strains.
ROS cellular level was quantified by fluorescence spectroscopy using 2,7-dichlorfluorescein-diacetate (DCFH-DA 100µM, D6883, Sigma-Aldrich), on bacteria grown in control condition (LB) or LB+paraquat (PQ at 2.5 mM) and harvested in exponential phase (OD = 0.6-0.7). Mean values from three independent experiments. Difference between the control and paraquat medium is significant for each strain (* t-test, $p < 0.05$). Difference between each strain for a given medium is not significant (N.S.). The increase in fluorescence levels was 2.9-fold (7005 vs 2374 arbitrary units) for the WT and 4.6-fold (9729 vs 2120 A.U.) for the *oxyR* mutant. No growth was observed for the *oxyR* mutant at a higher PQ concentration of 5mM (see Fig1).

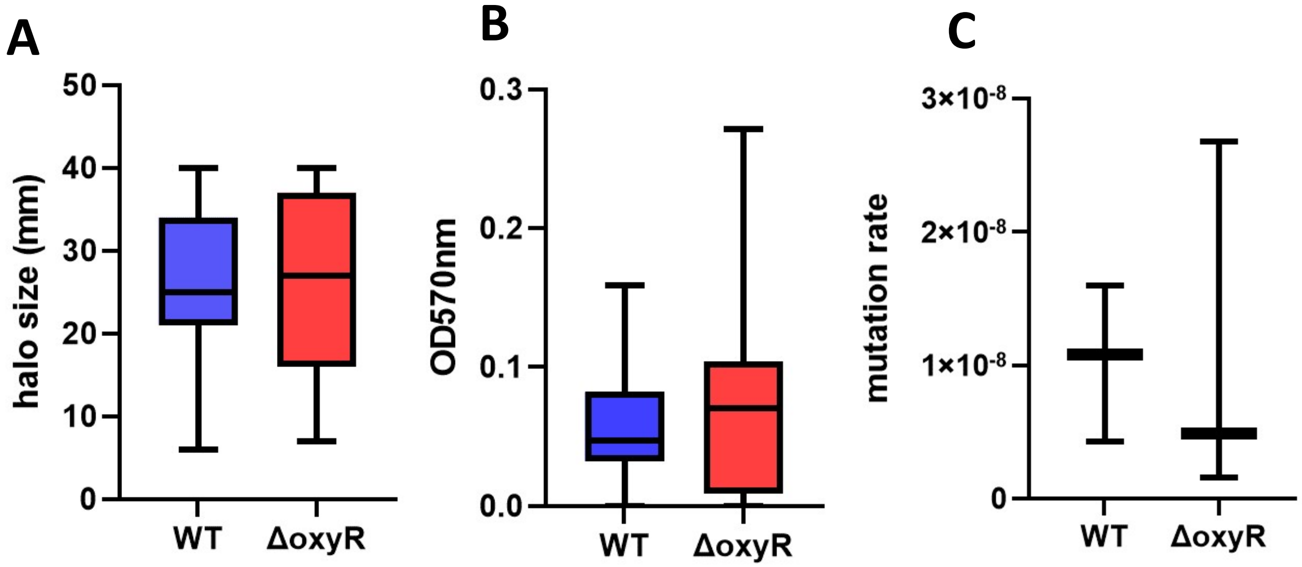

Fig. S2. Deletion of the *X. nematophila oxyR* gene does not modify motility, biofilm formation, nor mutation rate.
(A) Swimming motility. 0.35 % agar LB medium was inoculated using 5 µL of *X. nematophila* WT or DoxyR grown in exponential phase (OD_{600nm} = 0.8). The diameter of the halo size of swimming motility was measured 20 h after incubation. Data from 3 independent experiments (with 10 plates used in each condition) Differences are not significant (Wilcoxon test, p>0.05).
(B) Biofilm formation assays. Each strain was inoculated in 5 mL of LB in glass tubes and incubated four days at 28 °C without shaking. The amount of crystal violet staining (proportional to biofilm formation) was measured at $OD_{570.}$ Data from 3 independent experiments. Differences are not significant (Wilcoxon test, p>0.05).
(C) Mutation Rate. Spontaneous mutation rate was evaluated by quantifying the emergence of rifampicin-resistant CFUs. The two strains were grown overnight in 40 mL LB medium to reach a population above 1×10^9 CFU). Bacteria were serially diluted and plated on GNO or GNO with rifampicin (50 µg/mL). The mutation rate was calculated as the rifampicin-resistant population divided by the total population. Data from 3 independent experiments. Differences are not significant (t-test, p>0.05).

Fig. S3-A. Number of differentially expressed genes between the X. *nematophila OxyR* mutant and WT strain.
Venn diagram to compare the number of differentially expressed genes between *X. nematophila F1* and *oxyR* mutant, during LB-control (blue) or LB supplemented with paraquat (oxidative stress condition, red).

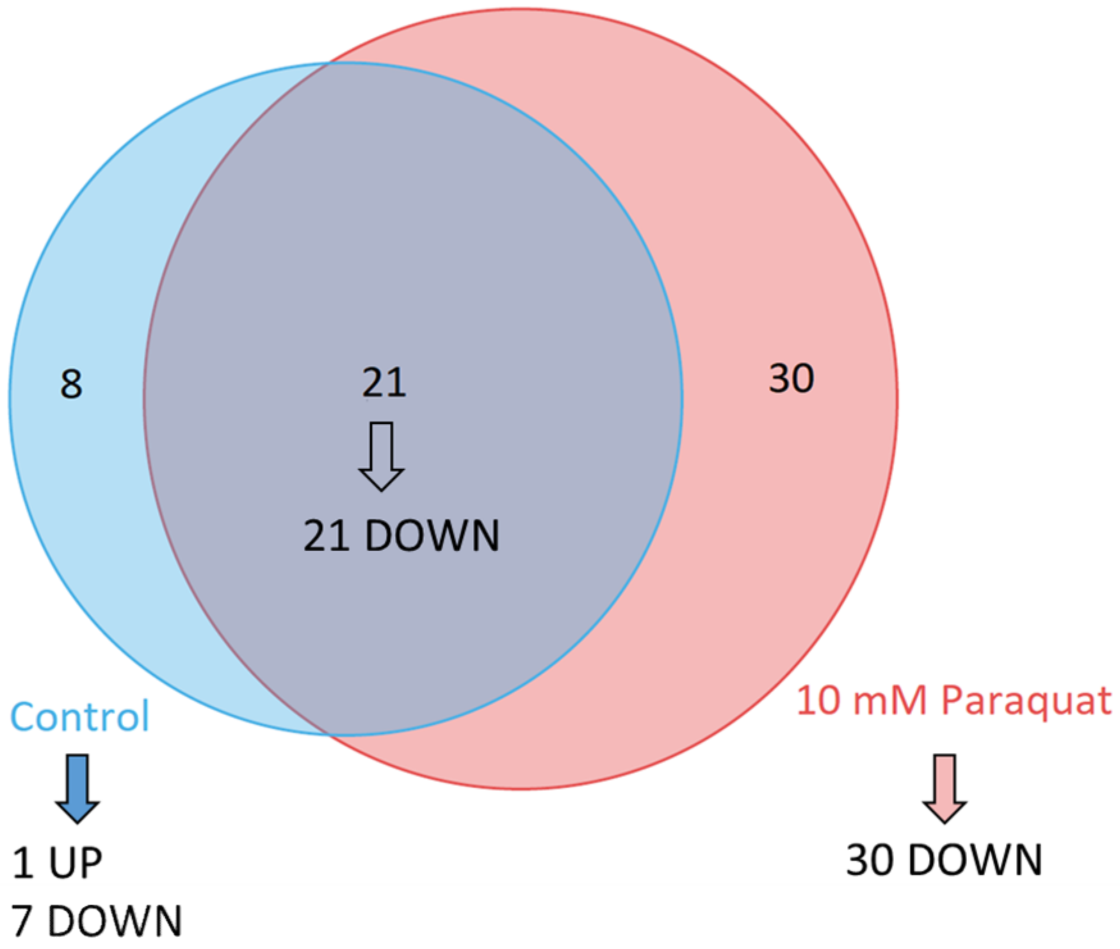

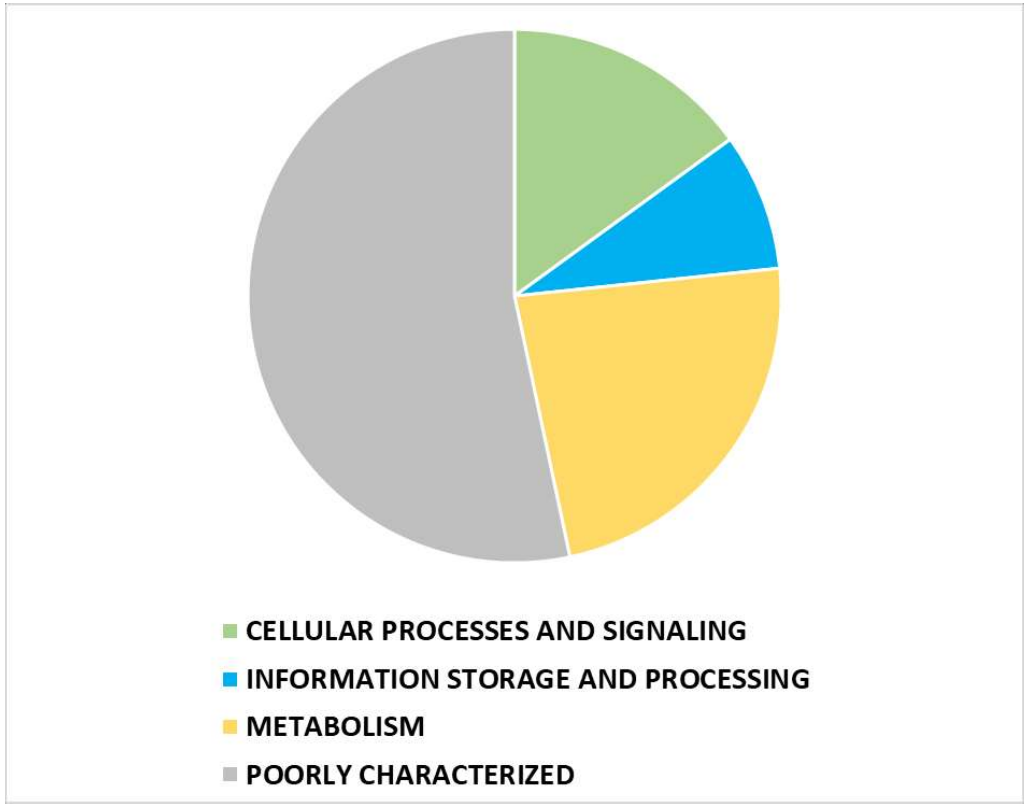

Fig. S3-B. Orthologous Group classification of the 59 differentially expressed genes between the *X. nematophila* WT and the *oxyR* mutant (EGGNOG Automatic Classification, see Method for details).

A

Gene	Product	LB		Paraquat 10 mM	
		RNAseq	RT-qPCR	RNAseq	RT-qPCR
		FC ΔoxyR/WT	FC ΔoxyR/WT	FC ΔoxyR/WT	FC ΔoxyR/WT
argH	argininosuccinate lyase	-	-	0,601	0,532
dps	Fe-binding and storage protein	-	-	0,105	0,107
trxB	thioredoxin reductase 1	-	-	0,774	0,782
pntA	pyridine nucleotide transhydrogenase, alpha subunit	-	-	0,343	0,230
yraJ	putative outer membrane protein	0,187	0,031	0,355	0,188
fimA	putative Fimbrial subunit (Pilin)	0,012	0,071	0,015	0,049
gor	glutathione oxidoreductase	-	-	0,250	0,211

B

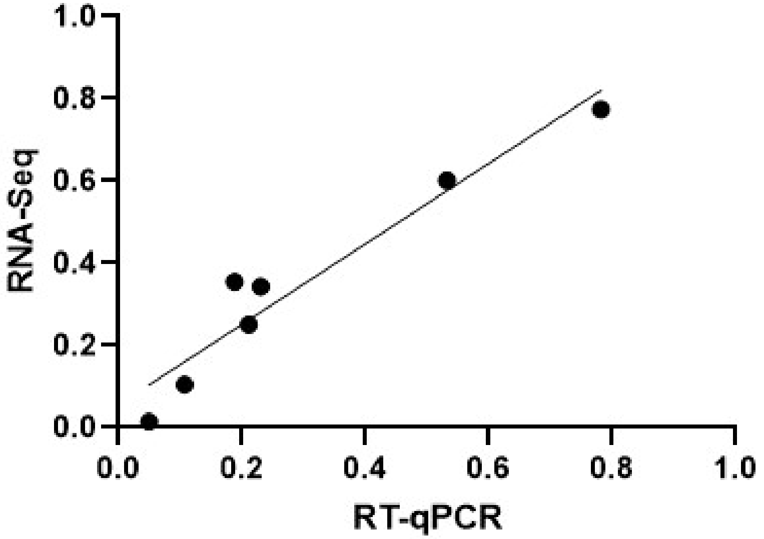

Fig. S4 Comparison between RT-qPCR and RNA-Seq data

(A) The fold change ratio (*oxyR* mutant/WT) obtained by RNASeq and the fold change ratio obtained by RT-qPCR is indicated for seven genes (*argH, dps, trxB, pntA, yraJ, fimA* and *gor*). “-”, NS

(B) The correlation factor (R^2) between RNAseq and RT-qPCR was calculated from the fold change ratio (*oxyR* mutant/WT) obtained by RNASeq against the fold change ratio obtained by RT-qPCR for the 7 genes tested. A strong positive correlation was reached using linear regression analysis (R^2 = 0.927).

S. carpocapsae / WT

S. carpocapsae / Δ*oxyR*

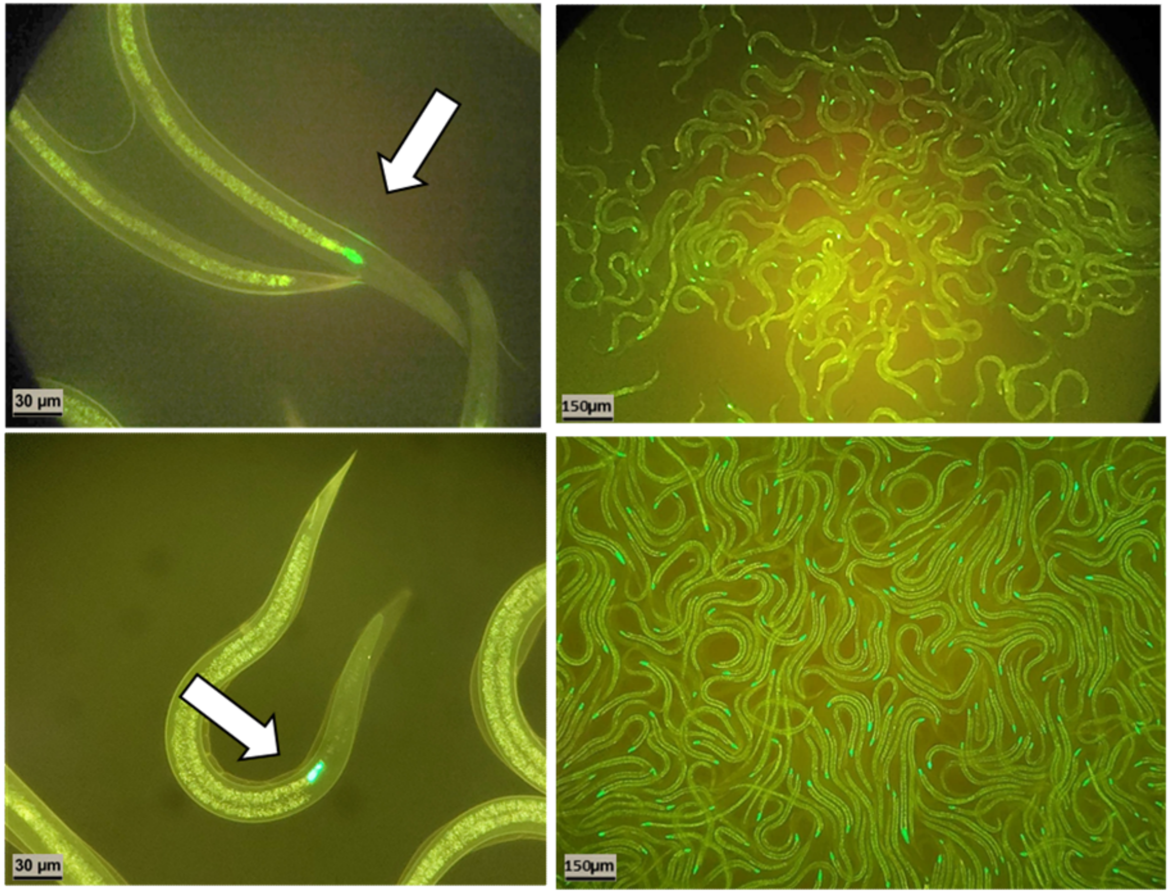

Fig. S5. Successful symbiotic reassociation between the *S. carpocapsae* nematode and the GFP-labelled *X. nematophila* bacterial strains.

Left, the presence of GFP-labelled bacteria in the receptacle of infective juvenile (IJs) stage of *S. carpocapsae* is marked by an arrow; right, all the observed IJs displayed a GFP-labelled receptacle.

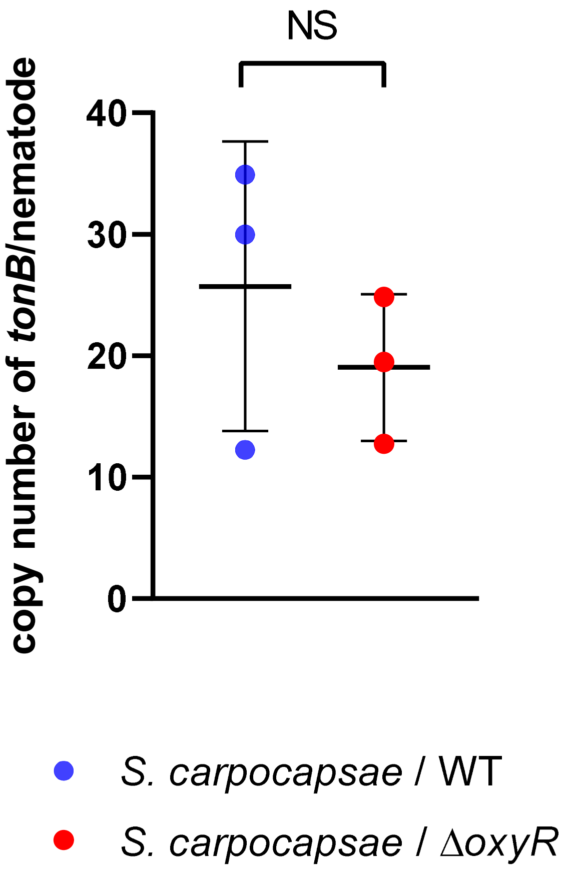

Fig. S6. Quantification by qPCR of the *X. nematophila* XNC1_0073 (TonB-dependent receptor) copy number per nematode *S. carpocapsae,* when associated with the WT (blue) or ΔoxyR (red) bacterial strains.
Differences are not significant (t-test, $p>0.05$).

Table S1: **Phenotypes of *X. nematophila* F1 WT and *oxyR* mutant strains**

Strain	Tested phenotypes [a]									
	Btb adsorption [b]	Licithinase activity [c]	Antibiotic production[d]	Sheep blood hemolysis[e]	Motility[f]	Lipolysis of[g]				
						Tween 20	Tween 40	Tween 60	Tween 80	Tween 85
WT	B	+	+	+	+	+	+	+	-	-
ΔoxyR	B	+	+	+	+	+	+	+	-	-

[a] All plates were incubated for 2 days at 28°C before assays were interpreted, unless otherwise indicated.

[b] Btb, bromothymol blue; B, dark blue colonies on NBTA medium.

[c] . production of a halo of precipitation surrounding each colony on Nutrient Agar plate containing 0.01% lecithin

[d] +, Halo size (>25 mm) of growth inhibition of *Micrococcus luteus*.

[e] +, halo of hemolysis detected

[f] +, Large spreading area (halo size > 15 mm) after 20h of incubation.

[g] +, Halo of precipitation; -, no halo of precipitation

[h] Growth rate was estimated on 4 independent biological replicates, using serial dilution of the inoculum as described in the method section. Differences were not significant (p=0.92, Student t-test). ND, not done.

Table S2: Oligonucleotides used in this study

Name	Sequence (5' to 3')	Description
upF-oxyR-Pst	TGAACTGCAGCCTGGATACATGGCTGGATT	construction of *oxyR* mutant
upR-oxyR-Sal	CGACTGGAAGTCGACATAATACCTTGCGGCTCGTG	construction of *oxyR* mutant
dnF-oxyR-Sac	TCGTGATACGAGCTCTGAGTTATCCGGCGAGAAG	construction of *oxyR* mutant
dnR-oxyR-Xba	TGGATCTAGACGCCCAATTCAAACATCTG	construction of *oxyR* mutant
Cm-F	AGGTATTATGTCGACTTCCAGTCGGGAAACCTG	construction of *oxyR* mutant
Cm-R	ATAACTCAGAGCTCGTATCACGAGGCCCTTTCG	construction of *oxyR* mutant
VerifDoxyR-F	ATCGGGTCACTTCTGGTTCTT	construction of *oxyR* mutant
VerifDoxyR-R	GCAACAACTGACAGCAATGAA	construction of *oxyR* mutant
Cp-oxyR -F	TACGAATTCATGCTGAGGTTGGGAATGAATATC	*oxyR* complementation
Cp-oxyR-R	GCATGGATCCAAAAGGGTGAAGGCAATAAT	
PoxyR-F-Xba	GCGTCTAGAGTTTTCTCTGCTTGAATT	construction of X. nematophila P*oxyR* -GFP fusion
PoxyR-R-Eco	CGGCGAATTCCAGCATTTGATATCAAT	
GFPRev	TCACCTTCACCCTCTCCACT	PCR verification of GFP-fusion
Pprobe F	AGGAATTGGGGATCGGAAG	
RT+_argC-Fw	CTCCGATATTTCTGGAAGCG	Reverse transcription PCR
RT+_argC-Rv	ATTGGTGATTGATGCCTTCC	
RT+_argB-Fw	AGCTAACAAGAAACTGCTGG	
RT+_argB-Rv	TTTTGCCCTTTACCATCCAG	
RT+_argG-Fw	AAAGTGCGTGATATTCCGAC	
RT+_argG-Rv	TTCTCCACAATGTCGATTCG	
RT+_argH-Fw	TTATGTGGAAATGCTGGCTC	
RT+_argH-Rv	TGGCATCAAAGAAGAACCAG	
RT+_oxyR-Fw	CTGCATATTGGCCTTATCCC	
RT+_oxyR-Rv	AGCATCAATAACTTCTCGCC	
RT-1-CB-Fw	TATCTGATCATTGTGGGTGC	
RT-1-CB-Rv	CCCCACCATGTACAATAACC	
RT-2-BG-Fw	GGACGGCATGATAGTGAAAG	
RT-2-BG-Rv	CACCCCTTCCAAATCTTCAC	
RT-3-GH-Fw	TCACGCTGAAGGTTTTATCC	
RT-3-GH-Rv	TCATTCAAAGCCAGTTCCAG	
RT-4-HR-Fw	TTGTCTGGATAAACGTCTGG	
RT-4-HR-Rv	ACGCCTAAATCATCCTCAAG	
qXn0509-argH_F	AAAGATCAGATTGCCCATATTCAGC	RT-qPCR
qXn0509-argH_R	CATTTCCACATAAGCAAGACACCAG	
qXn0510-oxyR_F	TGCTGAGGTTGGGAATGAATATC	
qXn0510-oxyR_R	TCACGCCTAAATCATCCTCAAGT	
qXn1716-dps_F	CAAAATTAATCAAAACCCCGCCTTC	
qXn1716-dps_R	TGAATAGAAATAAAATTCCGGCCCC	
qXn1779-trxB_F	GGAATATACCTGTGATGCGTTGATC	
qXn1779-trxB_R	CGGTGAATCAGATGAACTTCAGAAG	
qXn2490-pntA_F	AATATTGCGGGTTATCGTGCTATTG	
qXn2490-pntA_R	TGCTTTGTACTTGTTCCTTGACTTC	
qXn2826-yraJ_F	AGTCGCGTTGGGAAAATCTTAATAC	
qXn2826-yraJ_R	CGGCAGCATATTATCATCAGACATC	
qXn2828-fimA_F	TATCGCAGTAGTTATTTTGGGCTC	
qXn2828-fimA_R	CCTTTGGTTTAACATTACTTAGCCC	
qXn4548-gor_F	ACGATTATATTGCAATTGGTGGTGG	
qXn4548-gor_R	ATCAAAACCATAATCGGCACCATAC	
L-recA	TTAATACTCTGGGAGAGTTGATCG	
R-recA	GTTTCTTATTCAACTCAGCAGCAG	
mreBqf	AATTCTGGTGGGCACTGTTC	
mreBqR	GGATCGGCTTATCCAACAGA	
Xeno_F	ATGGCGCCAATAACCGCAACTA	qPCR on *X. nematophila* associated to nematodes
Xeno_R	TGGTTTCCACTTTGGTATTGATGCC	qPCR on *X. nematophila* associated to nematodes
63Bis	GAAGAGTTTGATCATGGCTC-	PCR 16S gene
153Rev	AAGGAGGTGATCCAGCCGCA	PCR 16S gene
